# Corticospinal Tract and Related Grey Matter Morphometric Shape Analysis in ALS Phenotypes: A Fractal Dimension Study

**DOI:** 10.3390/brainsci11030371

**Published:** 2021-03-14

**Authors:** Venkateswaran Rajagopalan, Erik P. Pioro

**Affiliations:** 1 Department of Electrical and Electronics Engineering, Birla Institute of Technology and Science Pilani, Hyderabad Campus, Hyderabad 500078, India; venkatnrp78@gmail.com; 2 Neuromuscular Center, Department of Neurology, Neurological Institute, Cleveland, OH 44195, USA; 3 Department of Neurosciences, Lerner Research Institute, Cleveland Clinic, Cleveland, OH 44195, USA

**Keywords:** amyotrophic lateral sclerosis, corticospinal tract, fractal dimension, grey matter, white matter

## Abstract

A pathological hallmark of amyotrophic lateral sclerosis (ALS) is corticospinal tract (CST) degeneration resulting in upper motor neuron (UMN) dysfunction. No quantitative test is available to easily assess UMN pathways. Brain neuroimaging in ALS promises to potentially change this through identifying biomarkers of UMN dysfunction that may accelerate diagnosis and track disease progression. Fractal dimension (FD) has successfully been used to quantify brain grey matter (GM) and white matter (WM) shape complexity in various neurological disorders. Therefore, we investigated CST and whole brain GM and WM morphometric changes using FD analyses in ALS patients with different phenotypes. We hypothesized that FD would detect differences between ALS patients and neurologic controls and even between the ALS subgroups. Neuroimaging was performed in neurologic controls (*n* = 14), and ALS patients (*n* = 75). ALS patients were assigned into four groups based on their clinical or radiographic phenotypes. FD values were estimated for brain WM and GM structures. Patients with ALS and frontotemporal dementia (ALS-FTD) showed significantly higher CST FD values and lower primary motor and sensory cortex GM FD values compared to other ALS groups. No other group of ALS patients revealed significant FD value changes when compared to neurologic controls or with other ALS patient groups. These findings support a more severe disease process in ALS-FTD patients compared to other ALS patient groups. FD value measures may be a sensitive index to evaluate GM and WM (including CST) degeneration in ALS patients.

## 1. Introduction

The pathological hallmarks of amyotrophic lateral sclerosis (ALS) are combined degeneration of the corticospinal tract (CST) and anterior horn cells, progressively affecting upper motor neurons (UMNs) and lower motor neurons (LMNs), respectively to result in classic ALS (ALS-Cl). Although electromyography objectively assesses LMN dysfunction, there is no clinically available test equivalent to easily detect UMN involvement [1,2,3]. On average, definitive diagnosis of ALS is delayed approximately 12 months after symptom onset supporting the need for biomarkers to shorten this interval. Neuroimaging in ALS has seen an unprecedented growth in recent years and has begun to characterize ALS phenotypes and provide insights into the disease process [4]. Potentially, identifying neuroimaging biomarkers of UMN in ALS could accelerate time to diagnosis, allow monitoring of disease progression and assist in assessing the efficacy of pharmacotherapies.

As early as 1909, Holmes [5] observed degeneration of the entire corticospinal system in ALS patients. More recently, conventional MRI studies using T2-[6], proton density-weighted and fluid attenuated inversion recovery (FLAIR) sequences, [7] have reported abnormal signal intensity along the CST. Depending on the study, bilateral CST hyperintensities are visible on conventional MRI scans in 17% to 67% (median 40%) of ALS patients with predominant UMN signs [8]. Some consider these qualitative findings to be nonspecific as they may occur in other disorders or even in some healthy individuals. However, while the aforementioned MRI sequences identify CST hyperintensity in some patients with UMN-predominant ALS (ALS-CST+), they do not in others (ALS-CST−). The reason for this difference is unknown. Recent quantitative MRI techniques, such as diffusion tensor imaging (DTI), have demonstrated CST degeneration in ALS patients [9,10,11]. A meta-analysis report based on multiple DTI studies of ALS brain revealed white matter (WM) degeneration not only in the CST but also in extramotor regions [12]. Previous DTI studies using region of interest (ROI) identification of the CST observed decreased fractional anisotropy (FA) values especially in the posterior limb of the internal capsule, where hyperintensity on conventional MRI is consistently reported in ALS patients compared to controls [1,9,10,13,14,15]. Use of ROI identification in these studies [9,10,14,15] limits analysis to only certain CST levels. To overcome this drawback, tensor based spatial statistics (TBSS) analysis of whole brain WM DTI data has been used. TBSS analysis has revealed WM degeneration in forceps minor, anterior corpus callosum, anterior inferior longitudinal fasciculus and CST of ALS patients with frontotemporal dementia (ALS-FTD) and behavioral variant FTD patients [16]. However, TBSS analysis is limited in that it is not an unbiased whole brain approach using diffusion tensor tractography (DTT). We previously demonstrated disruption or loss of DTT-reconstructed virtual CST fibers primarily in ALS-CST+ patients rather than in ALS-CST− patients [17]. In another recent study using DTT based graph theory network analysis [18], we found severe WM degeneration primarily in frontal and temporal lobes in ALS-FTD patients. These results indicate that DTT approach may be more robust when compared to ROI- or TBSS-based analyses.

Along with CST WM degeneration, another hallmark of ALS is degeneration of precentral gyrus or primary motor cortex (PMC) grey matter (GM). Structural neuroimaging has revealed significant cortical thinning and cortical surface area reduction in the PMC of ALS patients [19]. A previous study [20] comparing cortical thickness changes in ALS patients with classic signs, UMN predominant- or LMN predominant- phenotypes found that UMN predominant-ALS patients demonstrated more thinning of PMC than the other groups. A large population of ALS patients displayed significantly decreased cortical thickness not only in the PMC but progressive cortical thinning in parahippocampal and fusiform cortex during longitudinal follow-up [21]. Another imaging study using T2* sequences [22] found a higher signal hypointensity (caused by iron-laden reactive microglia) to thickness ratio of PMC in ALS patients compared to controls. All these results provide evidence of degenerating cortical GM that overlies CST WM tracts in ALS.

Axonal swelling with neurofilament accumulation, wallerian degeneration of axons and dendritic attenuation are some of the typical neurodegenerative changes seen microscopically in ALS [23]. If severe enough, the microscopic changes of axonal degeneration [24] may result in a reduction of WM complexity detected macroscopically. Fractal dimension (FD) is a quantitative approach that measures shape complexity of brain WM at the macroscopic level. Therefore, in this study we combine the two neuroimaging methods of DTT, which detects microscopic level changes, and FD, which detects macroscopic level shape changes to noninvasively examine microscopic and macroscopic brain pathology in patients with ALS.

FD has successfully identified brain WM complexity changes in aging [3,25] and in some non-ALS neurologic disorders; it has not yet demonstrated GM and CST degeneration in ALS. Studies in multiple sclerosis patients have found FD values to be increased in GM [26] and decreased in WM compared to controls [27]. FD analysis was superior to volumetric analysis in patients with multiple system atrophy of the cerebellar type and found significantly lower cerebellar WM and GM FD values than in controls [28]. Patients with mild Alzheimer’s disease have significantly different cortical ribbon FD values than do controls [29].

Our previous FD study [30] using T1-weighted (T1-w) brain images of ALS patients demonstrated global WM shape morphometric changes. Unlike DTI, T1-w imaging is qualitative, does not capture microscopic changes in brain tissue, and suffers from partial volume effects (GM-WM interface, cerebrospinal fluid [CSF]-WM interface) when segmenting WM tissue for FD shape analysis. DTI, on the other hand, is restricted to only WM voxels and provides more robust and more accurate FD measures in WM than does T1-w imaging of whole brain. Our recent DTI studies of the CST in ALS phenotypes [17,18] demonstrated that the DTT-based approach is highly sensitive in detecting WM pathology. To our knowledge, no FD studies of CST shape morphometric changes in ALS have been published. Using DTT to isolate specific WM fiber tracts (e.g., CST) from other brain WM would allow focused FD study of shape changes in such tracts. We also aimed to understand whether FD shape metric analysis of specific WM tracts could be a useful neuroimaging biomarker in ALS.

Therefore, the aim of this study was to apply FD analysis to investigate differences in structural degeneration of GM and WM CST arising from PMC and postcentral gyrus (primary sensory cortex, PSC) or PMC-CST and PSC-CST, respectively, in different ALS clinical phenotypes. We hypothesized that FD analysis is able to detect quantitative differences in shape morphometry between ALS patients and neurologic controls and between the ALS groups.

## 2. Methods

### 2.1. Demographics

Cleveland Clinic Institutional Review Board approved routinely acquired clinical MRI data to be stored and analyzed as de-identified images after obtaining the patient’s verbal consent. Data from 89 subjects were assigned into one of the following groups after evaluating their clinical presentations and MR images: (a) neurologic controls (mostly non-ALS mimics) whose clinical characteristics are shown in Supplementary Table S1, (b) ALS patients with classic (typical) UMN and LMN changes (ALS-Cl), (c) UMN-predominant ALS patients with CST hyperintensity seen on T2/PD-weighted images (ALS-CST+), (d) UMN-predominant ALS patients without CST hyperintensity on T2/PD-weighted images (ALS-CST−), and (e) ALS patients with frontotemporal dementia (ALS-FTD). The latter group of ALS patients were identified after bedside clinical cognitive screening (by Montreal Cognitive Assessment, MoCA) identified them as clinically impaired or demented and subsequent formal neuropsychometric testing; behavioral symptoms were noted, if present. Demographics of the above subjects and clinical measures of ALS patients are shown in Table 1.

### 2.2. Imaging Protocol

All sequences reported below are part of our routine ALS clinical MRI protocol obtained on a 1.5T Siemens Symphony (Erlangen, Germany) scanner. High resolution 3D T1-w axial MRI images of whole brain were obtained using magnetization-prepared rapid gradient echo (MPRAGE) sequence with these parameters: TR (repetition time) = 1800 ms, TE (echo time) = 4.38 ms, flip angle = 10°, inversion time (TI) = 1100 ms, slice thickness = 1 mm, in-plane resolution = 0.9 × 0.9 mm^2^, and number of slices = 160. T2- and PD-weighted images using a dual-echo FSE sequence were obtained with the following parameters: TR = 3900 ms, TE = 26 ms and 104 ms, echo train length or turbo factor = 7, number of averages = 1, slice thickness = 4 mm, in-plane resolution = 0.9 × 0.9 mm^2^, and number of contiguous slices = 40. DTI data were acquired by single shot echo planar imaging (SS-EPI) to obtain 12 diffusion-weighted (b = 1000 s/mm^2^) images and one b = 0 s/mm^2^ image. Imaging parameters included: TR = 6000 ms, TE = 121 ms, EPI factor = 128, resolution = 1.9 × 1.9 × 4 mm^3^, and number of slices = 30.

### 2.3. Data Processing

PD- and T2-weighted images were used primarily to assess for presence of CST hyperintensity, as part of our routine clinical evaluation of ALS patients.

### 2.4. White Matter Fractal Dimension Analysis

Diffusion tensor images were corrected for susceptibility artifacts using FSL’s FUGUE [31,32,33] and then for eddy current distortion effects. The b-matrix was rotated and stored [34,35]. Linear fitting of DTI was done using DTI Studio open software (https://www.nitrc.org/projects/mri_studio/, accessed on 5 August 2020), and DTT was performed using fiber assignment by the continuous tracking (FACT) algorithm [36]. Fiber tracking parameters were initiated from every voxel [37] with FA = 0, termination threshold set at 0.2, and track turning angle of 41°. After the above preliminary processing steps, Virtual CST fiber tracts on both sides were reconstructed after Wakana et al. [37] by placing the first ROI caudally in the cerebral peduncle. The second ROI rostrally was placed just beneath the PMC, as previously described [38] to identify the PMC-CST virtual fibers, or just beneath the PSC to identify the PSC-CST virtual fibers. Whole brain WM tracts were also obtained.

FD analysis of virtual fibers in PMC-CST (of left and right hemispheres), PSC-CST (of left and right hemispheres) and whole brain WM (excluding the CST fibers) was performed using 3D box-counting method [26,30,39] with our custom written MATLAB (version 2018b) code for automated processing. FD calculation steps included: (a) 3D MR images in nifti format were read into MATLAB using ‘niftiread’ command. (b) Box-counting method was used to calculate the fractal dimension as it can be applied to structures with or without self-similarity such as the human brain [3] employing the box-counting function in MATLAB (https://www.mathworks.com/matlabcentral/fileexchange/13063-boxcount, accessed on 6 August 2020). Briefly, the box-counting method applies boxes of different sizes = *r* on the given image and counts the number of boxes (*N*) needed to completely cover the entire binary image of the brain tissue. (c) FD is defined using power law, so log transformation was performed to linearize equation, as given below. (d) A linear regression fit of the log transformed equation shown below for different box sizes was estimated, and the FD estimate for the highest correlation value was considered.

ln*N* = FD ln(1/*r*) + ln*k*,

*k* = nuisance parameter.

DTT did not reconstruct all PSC-CST virtual fibers in 2 ALS-Cl patients, 3 ALS-CST+ patients, 2 ALS-CST− patients, and 1 ALS-FTD patient.

### 2.5. Grey Matter Fractal Dimension Analysis

Image processing steps for GM FD analysis included: (a) skull stripping of T1-w images using FMRIB Software Library (FSL) Brain Extraction Tool (BET) [33] (http://www.fmrib.ox.ac.uk/fsl/, accessed date 16 August 2020); (b) segment into WM, GM, and CSF probability maps using FSL’s FAST tool [40]; (c) register images to MNI152 space using the affine registration tool FLIRT [41,42] and then by nonlinear registration using FNIRT(www.fmrib.ox.ac.uk/analysis/techrep, accessed on 16 August 2020); (d) binarize GM probability maps; (e) obtain PMC and PSC ROIs in left and right hemispheres of each subject using the AAL atlas; (f) estimate FD values of these ROIs and of the whole brain GM (excluding PMC and PSC ROIs) of each subject using 3D box-counting method [39] with our custom written MATLAB (version 2018b) code, as described in the previous section.

### 2.6. Statistical Methods

Statistical comparisons of FD values between neurologic control and ALS groups, and between the ALS groups were performed using ANCOVA in SPSS (version 2020). As FD values are influenced by age [3], it was added as a covariate in the statistical model to exclude its effect. Correction for multiplicity to identify significant differences between patient groups was performed using the Least Significant Difference test (which does not adjust alpha for multiple comparisons) with *p* < 0.05. Demographics and clinical measures were compared between ALS patients using ANOVA with a significance level of *p* < 0.05.

### 2.7. Clinical Correlations

Relationships of FD values between ALSFRS-R, disease duration, El Escorial score, and disease progression rate were assessed using Spearman’s correlation analysis. As the El Escorial score (EES) is ordinal, it was converted into numeric form ranging from: possible ALS (EES = 1), probable with laboratory support ALS (EES = 2), probable ALS (EES = 3), to definite ALS (EES = 4).

## 3. Results

We thoroughly checked the FD values in all groups (predominantly in controls but also in all ALS patients) for any inexplicable differences in FD values between left and right brain hemispheres. We did not find such differences in FD values in either neurologic controls or ALS patients.

### 3.1. Fractal Dimension of Primary Motor Cortex-Originating Corticospinal Tract Fibers

Right hemisphere PMC-CST FD values in ALS-FTD patients were generally higher than in neurologic controls and other ALS groups. FD values were significantly higher in right hemisphere PMC-CST of ALS-FTD patients compared to ALS-CST− patients. Left hemisphere PMC-CST FD values showed no significant differences between neurologic controls and ALS patients or between ALS groups. These results are shown in Supplementary Figure S1.

### 3.2. Fractal Dimension of Primary Motor Cortex Grey Matter

Right hemisphere PMC GM FD values were significantly lower in ALS-FTD patients compared to neurologic controls, but no other group comparisons revealed significant differences. FD values in left hemisphere PMC GM showed no significant differences between neurologic controls and ALS patients or between ALS groups. These results are shown in Supplementary Figure S2.

### 3.3. Fractal Dimension of Primary Sensory Cortex-Originating Corticospinal Tract Fibers

**Figure 1:**
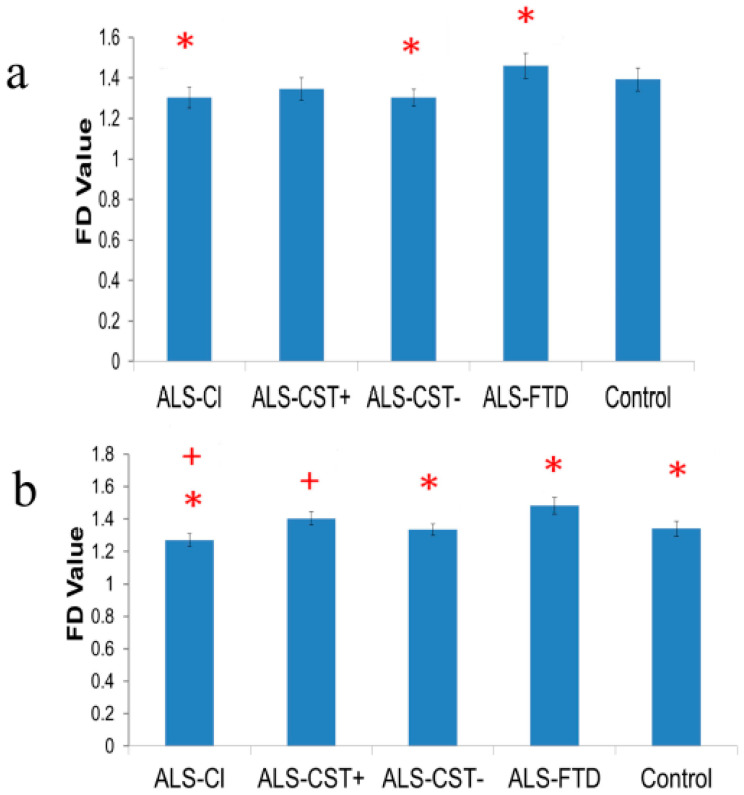
Compared to neurologic controls, FD values (±SEM) of PSC-CST are significantly lower in ALS-Cl and ALS-CST− patients but higher in ALS-FTD patients in right (**a**) and left (**b**) hemispheres. Significant differences are not detected between ALS-CST+ patients and neurologic controls but FD values are significantly lower in the left PSC-CST of ALS-Cl compared to ALS-CST+ patients, as indicated by ^+^ at *p* < 0.05. Significance between ALS-FTD and controls and other ALS subgroups at *p* < 0.05 is indicated by *.

ALS-FTD patients had significantly higher PSC-CST FD values than in ALS-Cl and ALS-CST− patients in both hemispheres (Figure 1), and in neurologic controls in the left hemisphere (Figure 1b). Of note, PSC-CST FD values were significantly lower in the left hemisphere of ALS-Cl compared to ALS-CST+ patients (Figure 1b).

### 3.4. Fractal Dimension of Primary Sensory Cortex Grey Matter

**Figure 2:**
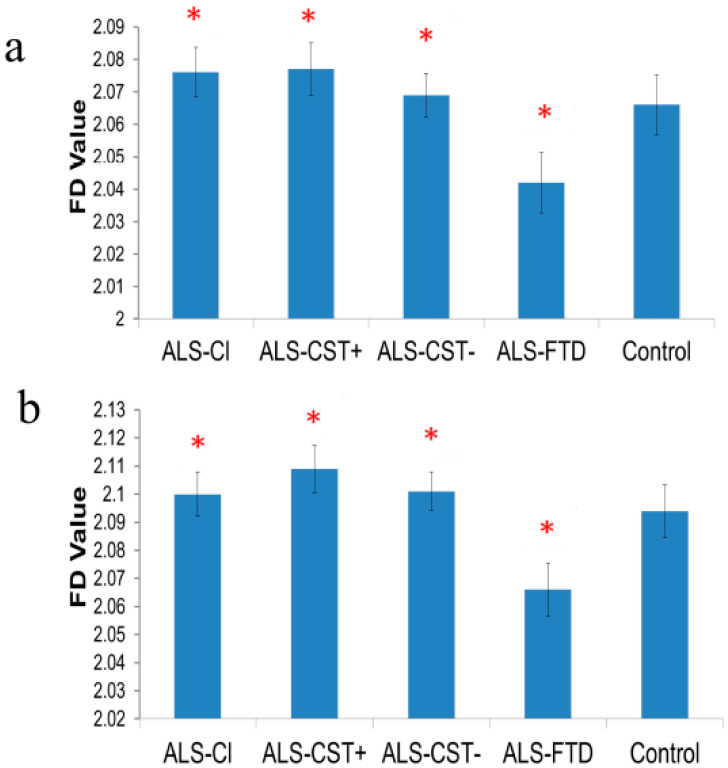
Compared to neurologic controls, FD values (±SEM) of PSC GM are significantly higher in ALS-CL, ALS-CST+, and ALS-CST− patients but lower in ALS-FTD patients of right (**a**) and left (**b**) hemispheres. * indicates significance between groups at *p* < 0.05.

FD values were significantly decreased in PSC GM of both hemispheres in ALS-FTD patients when compared to ALS-Cl, ALS-CST+, and ALS-CST− patients (Figure 2).

### 3.5. Fractal Dimension of Non-Primary Motor and Non-Primary Sensory Cortex Grey Matter

Significantly lower FD values were observed in whole brain GM (non PMC and non PSC) of ALS-FTD patients compared to neurologic controls and all other ALS groups. These results are shown in Supplementary Figure S3a.

### 3.6. Fractal Dimension of Non-Corticospinal Tract White Matter Fiber Tracts

No significant differences were noted in non PMC-CST and non PSC-CST whole brain WM tract FD values between neurologic controls and ALS patients or between ALS groups. These results are shown in Supplementary Figure S3b.

### 3.7. Correlation between Clinical and FD Measures

Before correcting for multiplicity, significant negative correlations were observed between FD values of whole brain GM (not including PMC and PSC) and of symptom duration (*r* = −0.33, *p* = 0.005) or disease progression rate (*r* = 0.25, *p* = 0.044). Similarly, before correcting for multiple comparisons, El Escorial scores correlated with FD value of right PMC-CST tracts (*r* = 0.31, *p* = 0.008). However, after correcting for multiple comparisons, no significant correlations were identified between any FD measures and ALSFRS-R scores, El Escorial scores, symptom duration, or progression rate.

### 3.8. Demographics and Clinical Measure Comparisons between Groups

ANOVA revealed significant differences (*p* < 0.05) in age, ALSFRS-R, duration of symptoms, and disease progression rate between groups. Chi-square Test found El Escorial scores to be significantly different (*p* < 0.05) between groups but not gender differences.

## 4. Discussion

The main goal of this study was to investigate brain shape morphometric changes revealed by FD values in GM, CST and non CST WM regions of ALS patients compared to neurologic controls. We wished to determine whether FD could be a useful imaging biomarker for ALS. The main findings of this study included: (a) significantly increased PMC-CST and PSC-CST WM FD values in ALS-FTD patients compared to other ALS groups, (b) significantly decreased PMC and PSC GM FD values in ALS-FTD patients compared to other ALS groups, (c) no significant FD value differences in other ALS groups compared to neurologic controls or between ALS groups.

FD analyses quantitatively assess shape morphometric features, i.e., complexity of brain WM and GM structures. No significant differences were observed in WM and GM FD values between neurologic controls and non-demented ALS patients or between the non-demented ALS groups. However, reductions were noted in left hemisphere PSC-CST FD values of ALS-Cl patients when compared to ALS-CST+ patients. Increased FD values in PMC-CST in the CST and PSC-CST of ALS-FTD patients suggest that neurodegeneration here significantly affects shape morphometry of the CST. Of note, ALS-FTD patients demonstrated significant changes in FD values of GM and WM tracts arising from PSC. This supports the understanding that neocortical neurons giving rise to the CST are situated not only in the PMC but also in adjacent cortical regions, including the PSC [43] and that subcortical WM fiber tract degeneration in ALS occurs in both motor and non-motor brain regions [44]. Furthermore, significant reductions of FD values in non-PMC and non-PSC GM without any subsequent significant FD change in underlying WM in ALS-FTD patients supports our previous findings [18] that neurodegeneration in such patients is predominantly a ‘neuronopathy’.

FD studies in multiple sclerosis (MS) [26,27] may provide insight into the underlying pathological mechanisms causing the abnormally high or low FD values in ALS-FTD patients. Reduced WM FD in MS compared to controls was proposed to arise from increased water content, decreased myelin content, and other inflammatory events causing abnormally amorphous tissue [27]. On the other hand, MS patients showed increased GM complexity [26] because of cortical inflammatory (e.g., microglial proliferation) and cellular changes (e.g., synaptic pruning, demyelination, blood–brain barrier alteration, etc.). Careful radiopathologic studies correlating MRI changes with same brain histopathology may reveal which pathologic changes contribute to the identified FD changes. A previous study of FTD brains suggested that GM atrophy and degeneration of WM tracts interconnecting such afflicted cortical regions disrupted the WM neuronal network, particularly in frontal and temporal lobes [45]. We have also found significant GM volume atrophy and reduced cortical thickness in frontal and temporal lobes of ALS-FTD patients (same population as in this study) compared to neurologic controls and other ALS groups [46]. Taken together, our results and the findings of Whitwell et al. [45] suggest that initial brain GM degeneration results in reduced WM structural complexity and accompanying dementia in ALS-FTD patients.

Absence of significant FD changes in other ALS phenotypes of ALS-Cl, ALS-CST+, and ALS-CST− suggests that either the disease process does not affect these measures at all or that it is insufficiently advanced (at the time of MRI) to alter the shape morphometry of CST or corticosensory WM/GM tissue. The latter explanation is more likely since WM abnormalities are already detected by DTI at this time in ALS-CST+ and ALS-CST− groups compared with neurologic controls [17,47]; CST shows reduced FA DTI values although no change in mean diffusivity (MD) [47,48]. Reduced FA may be attributed to axonal and myelin degeneration while unchanged MD values may represent gliosis restricting water molecule diffusion. It is presently unclear how WM FD—a metric of structural complexity at a macroscopic level—is affected by demyelination, axonal/wallerian degeneration, and gliosis. Progressive and cumulative microstructural degeneration (detected by DTI) likely precedes macrostructural changes (e.g., small fiber tract disruption) that eventually alter WM complexity (detected by FD). Changes in DTI metrics such as FA, axial diffusivity, and radial diffusivity reflect microscopic axonal and myelin damage. Such axonal and myelin damage in turn affects the macroscopic shape morphometry of fiber tracts, which we believe renders fiber tracts into more irregular and/or amorphous structures. Abnormal FD values of the WM fiber tracts in this study represent the macroscopic changes of brain WM occurring in ALS. Therefore, shape morphometry changes in ALS brain captured by FD metrics appear to be indirectly related to specific pathophysiology such as axonal or myelin damage, and gliosis or inflammatory processes.

Comparisons between the ALS groups revealed significant reductions in PSC-CST FD values of ALS-Cl patients when compared to ALS-CST+ patients. No other significant differences were observed in FD measures between any of the other ALS patient groups. Although this may reflect origin of descending motor CST fibers from the PSC, as discussed above, it may also represent degeneration of ascending sensory fiber tracts terminating in the PSC. MRI and neurophysiology-based studies have demonstrated sensory impairment in ALS [49,50]. Additionally, it is recognized that sensory impairment is clinically underestimated in ALS and that it may occur during early disease [51]. Our results of divergent FD changes along the PSC-CST: increasing in ALS-FTD patients and decreasing in ALS-Cl patients, suggest differential disease processes in these ALS groups. The significance and utility of FD detecting such morphometric changes is unclear but may serve to distinguish different clinical phenotypes of ALS.

Although no significant correlations between clinical and FD values were noted after correcting for multiple comparisons, we observed a trend towards significant positive correlations between FD measures of whole brain GM (excluding PMC and PSC) and disease progression rate. This suggests that higher FD values may identify ALS patients with faster disease progression. Positive correlations of El Escorial score with right CST tract FD values suggest that FD of CST may be a useful biomarker for early diagnosis of ALS.

At time of MRI, ALSFRS-R scores were lowest in ALS-FTD patients indicating greater functional impairment than in other ALS patients. Relatively similar ALSFRS-R scores between the other ALS groups (except of ALS-CST+ patients) suggested little difference in functional impairment and possibly disease stage. However, the significant differences between ALS groups of disease duration, progression rates, and El Escorial scores reflect the variable nature of the underlying neurodegenerative process. These observations support the value of studying ALS patients segregated into phenotypic groups and suggest that pathogenic mechanisms may differ in patients between such groups.

To our knowledge this is the first study quantifying shape morphometry changes of GM, CST- and non CST-related WM in different ALS phenotypes. As FD values are measured in log scale, even small differences between groups (seen in Figure 1 and Figure 2, Supplementary Figures S1–S3) reflect relatively large changes in GM and WM shape complexity. Future longitudinal studies of FD changes over the course of disease will shed more light on the potential role of FD as an imaging biomarker of ALS and disease progression. Considering the DTI data were acquired at 1.5T as part of our routine clinical MRI protocol with 12 diffusion-weighted directions, future validation studies should be performed at 3T with a higher direction number for higher resolution of virtual WM tracts. Analyses correlating FD values with histopathology in post mortem brains of same ALS patients (who underwent premortem MRI) may reveal tissue changes underlying shape morphometry and provide further insights into its relevance.

## 5. Conclusions

Of the four ALS phenotypes studied here, ALS-FTD patients have the greatest brain WM structural degeneration as revealed by FD, which appears to be a sensitive indicator of CST breakdown. The extent of GM and WM degeneration in ALS appears to be phenotype-dependent. Why the ALS disease process as revealed by FD is most severe in ALS-FTD patients compared to other patient groups is unknown and warrants further study.

## Figures and Tables

**Table 1:** Participant characteristics.

	ALS-Cl	ALS-CST+	ALS-CST−	ALS-FTD	Controls	Significance
Demographics						
Age (year)	57.5 ± 12.2	51.7 ± 11.6	59.4 ± 10.5	67.4 ±10.2	51.7 ± 15.7	*p* = 0.007
*n*	19	17	25	14	14	
Gender	11 male, 8 female	12 male, 5 female	15 male, 10 female	3 male, 11 female	9 male, 5 female	*p* > 0.05
ALSFRS-R	37.0 ± 9.3	33 ± 7.8	35.9 ± 7.0	29 ± 6.9		*p* = 0.047
Symptom duration (months)	27.2 ± 26.5	15.4 ± 8.1	61.4 ± 61.5	38.4 ± 20.5		*p* = 0.003
Disease progression rate	−0.7 ± 0.8	−1.5 ± 1.8	−0.4 ± 0.3	−0.6 ± 0.30		*p* = 0.009
El Escorial Score	2.5 ± 0.9	1.8 ± 1.1	1.4 ± 0.8	2.3 ± 1.3		*p* < 0.01

Disease progression rate is calculated as given in equation below:$\mathrm{Disease}\;\mathrm{progression}\;\mathrm{rate}=\frac{\left(48-\mathrm{ALSFRS}-R\;\mathrm{at}\;\mathrm{MRI}\right)}{\mathrm{Duration}\;\mathrm{of}\;\mathrm{symptoms}\;\left(\mathrm{months}\right)}$.

## Data Availability

Because the data are property of Cleveland Clinic, they cannot be shared and are not publicly available.

## Supplementary Materials

The following are available online at https://www.mdpi.com/2076-3425/11/3/371/s1, Table S1, Figure S1, Figure S2, Figure S3.
