# Supplementary material for: Corticospinal Tract and Related Grey Matter Morphometric Shape Analysis in ALS Phenotypes: A Fractal Dimension Study
Source: Brain Sci. 2021 Mar 14;11(3):371. doi: 10.3390/brainsci11030371 (PMC8001972; doi:10.3390/brainsci11030371)
Supplement: Supplementary file 1 [file brainsci-11-00371-s001.pdf]

Supplementary Material

Corticospinal Tract and Related Grey Matter Morphometric Shape Analysis in ALS Phenotypes: A Fractal Dimension Study

Venkateswaran Rajagopalan and Erik P. Pioro

Table S1. –Clinical characteristics of neurological controls.

Subject	Clinical condition
1	Severe fatigue
2	Stiff person syndrome with severe spasticity
3	Parkinsonism
4	Depression, cervical cancer, fibromyalgia
5	Fibromyalgia-like or myofascial pain syndrome
6	Painful sensory polyneuropathy
7	Insomnia from involuntary muscle twitches
8	Parkinson's disease or parkinsonism
9	Lower motor neuron abnormalities at cervical and lumbosacral levels
10	Non-length dependent small fiber sensory neuropathy
11	Pain in joint, pain in lower leg
12	Small fiber neuropathy
13	Large fiber neuropathy
14	Fibromyalgia-like syndrome

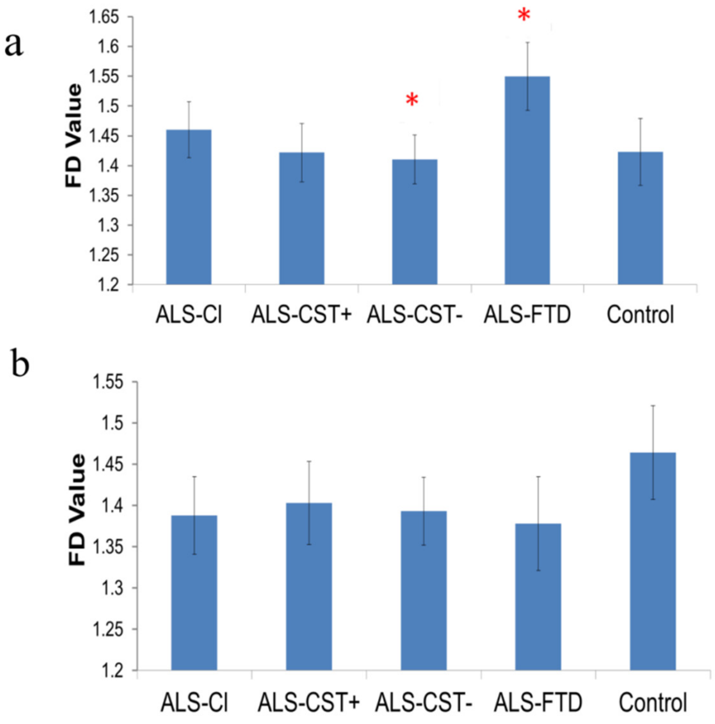

Figure S1. - Compared to neurologic controls, FD values (±standard error of mean, SEM) of PMC-CST are significantly decreased in ALS-CST- patients and increased in ALS-FTD patients of only the right (a) but not left (b) hemisphere. *indicates significance between groups at $p < 0.05$.

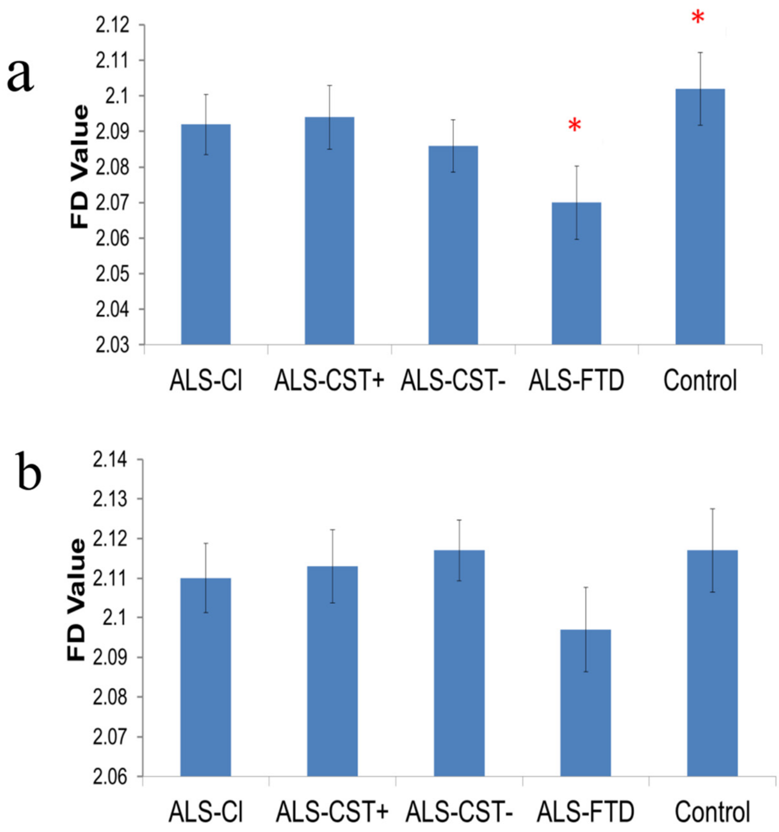

Figure S2. - Compared to neurologic controls, FD values (±SEM) of PMC GM are significantly decreased in right (a) but not left (b) hemisphere of only ALS-FTD patients. *indicates significance between groups at $p < 0.05$.

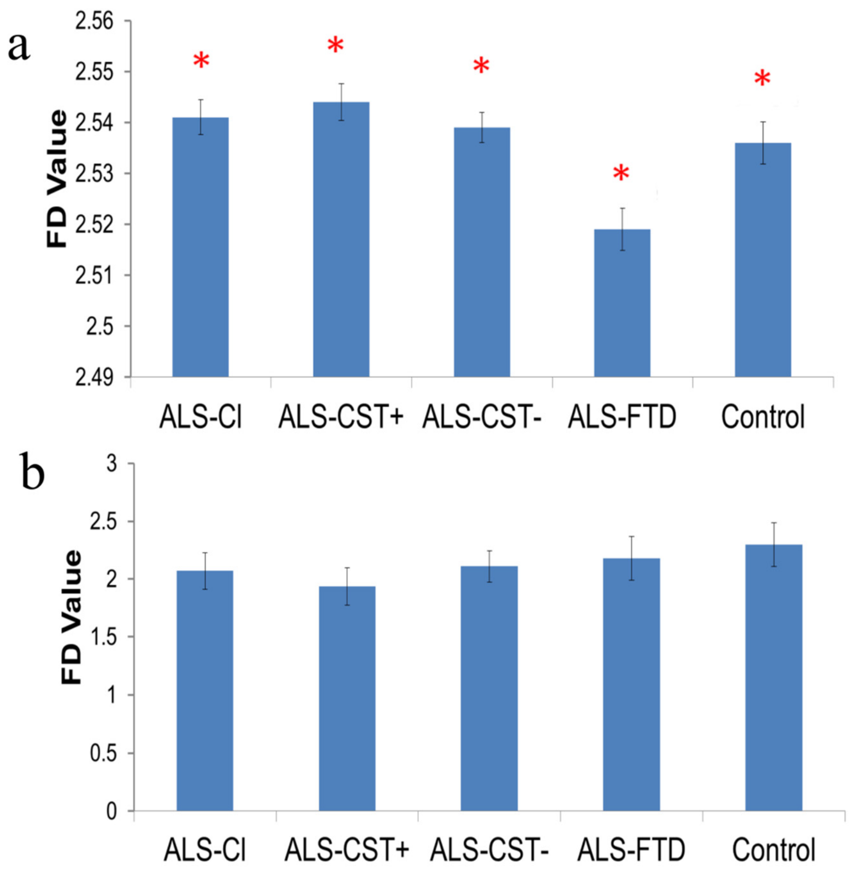

Figure S3. - Compared to neurologic controls, FD values (±SEM) of non-PMC and non-PSC GM in (a) are increased in ALS-Cl, ALS-CST+, and ALS-CST– patients but decreased in ALS-FTD patients. However, FD values (±SEM) of non-CST WM in (b) are not significantly different between neurologic controls and ALS groups. *indicates significance between groups at $p < 0.05$.
